# High Glucose Suppresses Keratinocyte Migration Through the Inhibition of p38 MAPK/Autophagy Pathway

**DOI:** 10.3389/fphys.2019.00024

**Published:** 2019-01-28

**Authors:** Lingfei Li, Junhui Zhang, Qiong Zhang, Dongxia Zhang, Fei Xiang, Jiezhi Jia, Ping Wei, Jiaping Zhang, Jiongyu Hu, Yuesheng Huang

**Affiliations:** ^1^Institute of Burn Research, Southwest Hospital, Third Military Medical University (Army Medical University), Chongqing, China; ^2^State Key Laboratory of Trauma, Burns and Combined Injury, Southwest Hospital, Third Military Medical University (Army Medical University), Chongqing, China; ^3^Endocrinology Department, Southwest Hospital, Third Military Medical University (Army Medical University), Chongqing, China; ^4^Department of Plastic Surgery, Southwest Hospital, Third Military Medical University (Army Medical University), Chongqing, China

**Keywords:** autophagy, p38/MAPK, cell migration, high glucose, diabetes

## Abstract

Wound healing is delayed frequently in patients with diabetes. Proper keratinocyte migration is an essential step during re-epithelialization. Impaired keratinocyte migration is a critical underlying factor responsible for the deficiency of diabetic wound healing, which is mainly attributed to the hyperglycemic state. However, the underlying mechanisms remain largely unknown. Previously, we demonstrated a marked activation of p38/mitogen-activated protein kinase (MAPK) pathway in the regenerated migrating epidermis, which in turn promoted keratinocyte migration. In the present study, we find that p38/MAPK pathway is downregulated and accompanied by inactivation of autophagy under high glucose (HG) environment. In addition, we demonstrate that inactivation of p38/MAPK and autophagy result in the inhibition of keratinocyte migration under HG environment, and the activating p38/MAPK by MKK6(Glu) overexpression rescues cell migration through an autophagy-dependent way. Moreover, diabetic wound epidermis shows a significant inhibition of p38/MAPK and autophagy. Targeting these dysfunctions may provide novel therapeutic approaches.

## Introduction

Diabetes mellitus (DM) is a common disease originated from poor glucose control. Impaired wound healing is one of the common and potentially serious complications and is still a main cause of morbidity and mortality in patients with diabetes (Robbins et al., 2008; Kilic et al., 2015; Zhang et al., 2015). The chronic complications in patients with diabetes are mainly attributed to the disruption of glucose homeostasis and increased glucose levels (Long et al., 2016). With the worldwide increasing prevalence of diabetes, uncovering the underlying molecular mechanisms that are responsible for the poor diabetic wound healing is a crucial public health issue.

Normal wound healing is a dynamic, interactive biological process involving inflammation, re-epithelialization and tissue remodeling. Re-epithelialization is an important step in the process of wound healing and is regulated by keratinocyte migration and proliferation (Shaw and Martin, 2016). Keratinocyte migration appears to be the most important cellular activity for re-epithelialization step to cover the denuded wound surface. Accumulating evidences have revealed that dysfunction of epidermal keratinocytes is a potentially crucial factor that are responsible for the poor healing of diabetic wounds (Hu and Lan, 2016). Although there are series of studies on wound healing in diabetes, little is known about the underlying molecular mechanisms involved in the impaired keratinocyte migration.

Macroautophagy (hereafter abbreviated as autophagy) is a highly conserved catabolic process featured by the formation of intracellular double-membrane structures that degrade and recycle misfolded proteins and damaged organelles (Maiuri et al., 2007; Gomes et al., 2016; Rybstein et al., 2018). Previously, autophagy in keratinocytes has been indicated as a regulator of early differentiation (Moriyama et al., 2014; Akinduro et al., 2016), a mechanism of senescent cell death (Gosselin et al., 2009; Deruy et al., 2014), and a prosurvival mechanism that protects from apoptosis induced by UV radiation (Moriyama et al., 2014, 2017). Growing evidences have revealed that autophagy is implicated in cell migration, although its exact role in cell migration is controversial (Sharifi et al., 2016; Sun et al., 2018). To date, little is known that whether the autophagy in keratinocytes is responsible for their migration in diabetic wound healing.

As is widely known that p38/mitogen-activated protein kinase (MAPK) is an important kinase responsible for cytoskeleton reorganization that promotes migration and proliferation in various cell types (Huang et al., 2004; Spindler et al., 2015). We and others have reported that p38/MAPK regulated keratinocyte migration under hypoxia (O’Toole et al., 2008; Woodley et al., 2009; Jiang et al., 2014; Guo et al., 2015). MAPKs are important mediators of autophagy upon various stresses, such as hypoxia, oxidative stress, and radiation. In addition, some studies have revealed that the activation of p38/MAPK was altered under high glucose (HG) environment (Kim and Choi, 2015). Therefore, it is potential that autophagy controlled by p38/MAPK regulates keratinocyte migration in diabetic wound healing.

In the present study, we found that keratinocyte migration was inhibited significantly under HG treatment. We also revealed that inactivated p38/MAPK and autophagy were responsible for the inhibition of keratinocyte migration. Activating p38/MAPK by MKK6(Glu) overexpression rescued cell migration through an autophagy-dependent manner. Together, our data provide a possible explanation for the impaired re-epithelialization in cases that levels of blood glucose are increased, such as diabetes.

## Materials and Methods

### Ethics Statement

All the described experiments here were conducted according to the Declaration of Helsinki Principles, and were authorized by the Animal Experiment Ethics Committee of the Third Military Medical University in Chongqing, China.

### Cells Culture

Human immortalized keratinocyte HaCaT cells were obtained from Cell Bank of the Chinese Academy of Sciences in Beijing, China and incubated in RPMI 1640 medium (SH30809, Hyclone) supplemented with 10% fetal bovine serum (10100139, Gibco), 100 U/ml penicillin, and 100 μg/ml streptomycin (Beyotime, China). Primary mouse keratinocytes (MKs) were cultured as previously reported (Jiang et al., 2013). Briefly, MKs were isolated from epidermis separated from the dermis by an overnight dispase treatment (4°C) of newborn C57BL/6J mice by 0.25% trypsin/0.04% EDTA solution (Invitrogen, United States) then cultured in keratinocyte serum-free medium (K-SFM medium) (Gibco, United States). The keratinocytes were incubated at 37°C, 5% CO_2_, and 95% humidity.

### HG Treatment

Previously, the subcutaneous tissue glucose concentration was found close to that in the plasma (Thomas et al., 1998), which is about 5.8 mM (Lan et al., 2008). Thus, it is reasonable to use 5.5 mM as the level of normal glucose (NG) in our study. Most *in vitro* studies applied 25 mM as HG concentration, thus it is suitable to apply glucose concentrations of 10, 15, and 25 mM for the hyperglycemic conditions in this study. A p38 inhibitor [SB203580 (SB), Selleck, 5 μm] was used and incubated at 37°C for 30 min prior to indicated treatments. 3-Methyladenine (3-MA) (Sigma, 5 mM) and bafilomycin A1 (BafA1, 10 nM, B1793, Sigma) were added to inhibit autophagy and kept in the keratinocytes with or without HG treatment.

### Cell Proliferation Assay

Cell proliferation was assessed by the Cell counting kit-8 (CCK-8; Beyotime) and was performed according to the manufacturer’s instructions. The 96-well plates were pre-incubated in a humidified incubator with 5% CO_2_ at 37°C for 24 h before CCK-8 solution was added to the plate. The plate was then incubated for another 2 h. The absorbance was measured at 450 nm using a microplate reader (Thermo Fisher Scientific, United States).

### Scratch Wound Healing Assay

Monolayers of keratinocytes cultured in 12-well plates were wounded by a 10-μl plastic pipette tip after being incubated at 37°C for 2 h with mitomycin-C (S8146, Selleck, final concentration: 5 μg/ml) to inhibit cell proliferation, and then rinsed with medium to remove any cell debris (Zhang et al., 2017). The wound healing process was monitored with an inverted light microscope (Olympus, Japan). Cell migration was defined as the wound-closure rate (%), which was analyzed using NIH ImageJ software^1^.

### Single Cell Motility Assay and Quantitative Analysis

Keratinocytes were seeded into 24-well plates at a density of 0.5 × 10^4^/cm^2^ in corresponding culture medium. Then time-lapse imaging was performed with a Zeiss imaging system (Carl Zeiss Meditec, Jena, Germany) with a CO_2_- and temperature-controlled chamber. The images were taken every 3 min for 3 h. Later, cells’ trajectories were obtained through tracing the position of cell nucleus at frame intervals of 6 min using NIH Image J software, and velocity (μm/min) of each cell was defined as the total length (μm) of the trajectories dividing by time (min), which reflected the capacity of cell motility.

### Recombinant Adenovirus Construction and Transduction

The recombinant adenovirus that constitutively activates MAPK kinase 6 [MKK6(Glu)], which specifically and steadily activates p38/MAPK signaling, was generated by Shanghai GeneChem, Co. Ltd (Shanghai, China).

### Small Interfering RNA (siRNA) Transfection

For RNA interfering, cells were transfected with siRNA specific for Atg5 (siAtg5) or corresponding scramble-siRNA (siNC) with lipofectamine 2000 (11668027, Invitrogen) according to the manufacturer’s protocol. The siRNAs were purchased from GenePharma Company (Shanghai, China).

### Induction of Diabetes Using Streptozotocin (STZ)

After 12 h fasting, C57BL/6J mice (aged 12–14 weeks) were injected with a single intraperitoneal dose of streptozotocin (S0130, Sigma) in saline at 150 mg/kg body weight. Body weight and random blood glucose concentration were monitored weekly after STZ injection until a diabetic state was verified. Mice with a glucose concentration exceeding 16.7 mmol/l were considered diabetic. Full-thickness dorsal wounds (5 mm in diameter) were performed 5 weeks post induction of diabetes and collected 7 days post wounding. Random blood glucose was measured using blood glucose strips and the glucometer (Abbott Diabetes Care Limited, United Kingdom).

### Western Blot Analysis

Whole cell extracts and mouse skin specimens were prepared in the RIPA lysis buffer for Western blot (P0013, Beyotime) and centrifuged at 14,000 rpm for 15 min at 4°C. The supernatants were then obtained and protein concentrations were detected using Bradford Protein Quantification Kit (500-0205, Bio-Rad Laboratories). The protein samples were loaded and separated by SDS-PAGE then transferred to PVDF membrane (Millipore). Membranes were incubated overnight at 4°C with specific primary antibodies. Sequentially, membranes were incubated with secondary antibodies and visualized using ChemiDoc XRS System (Bio-Rad Laboratories). Primary antibodies used for immunoblotting were as follows: LC3B (L7543, Sigma), Atg5 (12994, Cell Signaling Technology), p38 (8690, Cell Signaling Technology), phosphorylated p38 (p-p38; 4511, Cell Signaling Technology), and β-Actin (ab8227, Abcam).

### Immunoprecipitation (IP)

To discern the protein interaction between p-p38 and Atg5, whole cell extracts were prepared in the cell lysis buffer for Western blot and IP (Beyotime, P0013) and centrifuged at 14,000 ×*g* for 15 min. The supernatants were incubated with 2 μg of anti-p-p38 (4511, Cell Signaling Technology), anti-Atg5 Atg (12994, Cell Signaling Technology) for 8 h at 4°C, and then precipitated with Protein A/G Plus-Agarose (Santa Cruz) overnight at 4°C. Total and binding proteins were detected by western blotting. In addition, when performing IP, the denatured IgG heavy chain of the primary antibody used for IP runs at approximately 50 kDa on the subsequent western blot, which can obscure bands of Atg5 protein (55 kDa). An IgG light-chain specific secondary antibody was used to eliminate this problem.

### Immunofluorescence Staining

After treatments, samples (cells cultured on glass coverslips, and frozen sections of mouse skin) were fixed with 4% paraformaldehyde for 20 min after being rinsed twice with phosphate buffer saline (PBS). Subsequently, the samples were incubated with the primary antibodies at 4°C overnight, washed three times with PBS, and then stained with fluorescent secondary antibodies at 37°C for 1 h. Nuclei were counterstained with 4′, 6-diamidino-2-phenylindole (DAPI) (HyClone, United States) before imaging. The pictures were acquired using a Leica Confocal Microscope (Leica Microsystems, Wetzlar, Germany). The primary antibody LC3B (L7543, Sigma) and the secondary antibody Alexa Fluor 488 (Invitrogen, A32731 rabbit) were used in the present study.

### Statistical Analysis

All results were expressed as mean ± SEM. Comparisons between two groups were performed using a two-tailed unpaired *t*-test. Statistical significance among three or more groups were performed by a one-way analysis of variance (ANOVA). *P* < 0.05 was considered to be significant.

## Results

### HG Treatment Reduces Keratinocyte Migratory Capacity

**FIGURE 1:**
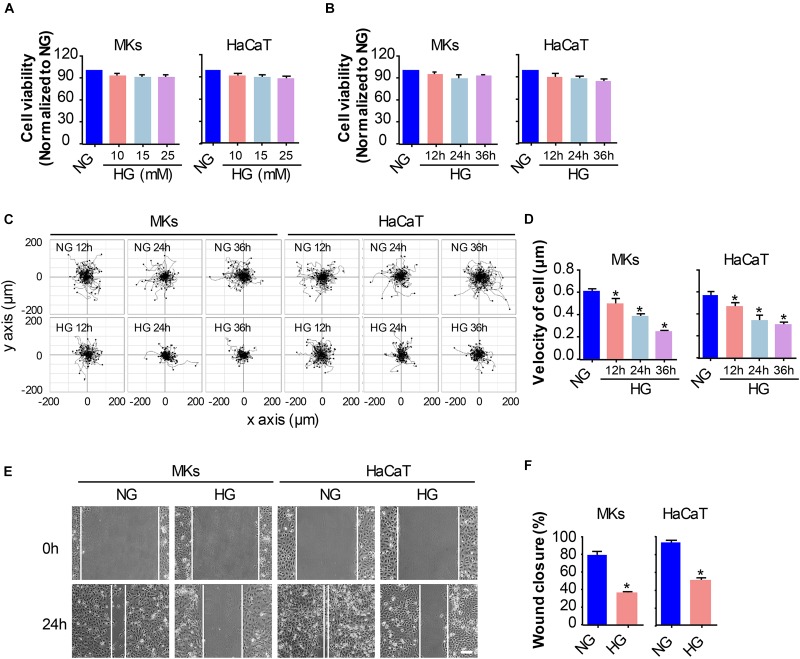
HG treatment reduces keratinocyte migratory capacity. **(A)** Keratinocytes (MKs and HaCaT cells) were subjected to various concentrations of glucose (10, 15, and 25 mM) for 24 h prior to the detection of cell viability using the CCK-8 assay. Results were shown as means ± SEM (*n* = 5). **(B)** A time-lapse experiment was set out to analyze the influences of 25 mM glucose on keratinocyte viability as determined by CCK-8 assay. Data were shown as means ± SEM. Then we assayed the effects of HG on keratinocyte mobility, and performed the single cell motility assay and the scratch wound healing assay. **(C)** Single cell motility assays were performed to detect the motility under HG. Representative images of cell trajectories were shown (*n* = 5). **(D)** Graph quantifying the average velocity of cell movement. Results were shown as means ± SEM. **(E)** Scratch wound healing assays were performed to detect the migration of indicated cells. Pictures of the scratched wounding were taken after 24 h culturing with or without HG treatment. Representative pictures of the scratched wound were shown (*n* = 5). Scale bar = 100 μm. **(F)** Graph quantifying the rate of wound closure. Results were shown as means ± SEM. ^∗^*P* < 0.05 vs. NG group. All the experiments were repeated three times.

Firstly, the keratinocytes (MKs and HaCaT cells) were subjected to different concentrations of glucose for 24 h prior to the detection of cell viability using the CCK-8 assay. As shown in Figure 1A, the viability of keratinocytes cultured in medium containing 5.5 mM glucose was served as the control (100%) for comparison. Treatment with 10, 15 and 25 mM glucose did not notably affect keratinocyte viability. Then, a time-lapse experiment was set out to analyze the influences of 25 mM glucose on keratinocyte viability (Figure 1B). Results showed little effects of 25 mM glucose on keratinocytes viability up to 36 h. These observations excluded the possibility that differences in the capability of keratinocyte migration were attributed to the alterations in cell viability.

To investigate the influences of HG on keratinocyte migration, we performed two cell migration assays, i.e., the single cell motility assay and the scratch wound healing assay. As shown in Figure 1C,D, results from the single cell motility assays demonstrated a remarkable decrease in the range of cell trajectory and the velocity of cell movements under 25 mM glucose treatment in a time dependent way as compared to that under normal concentration of glucose (NG, 5.5 mM). To confirm the above results, we performed the scratch wound healing assay, which has been indicated as an *in vitro* model to mimick the migration of keratinocyte during wound repair, to explore the influences of HG on keratinocyte migration. The wound closure in the monolayer keratinocytes exposed to HG (25 mM, 24 h) reduced significantly as compared to NG group (Figure 1E,F). These results indicated that HG treatment notably downregulated the migratory capacity of keratinocytes.

### HG Treatment Inhibits Autophagy and p38/MAPK Activity in Keratinocytes

**FIGURE 2:**
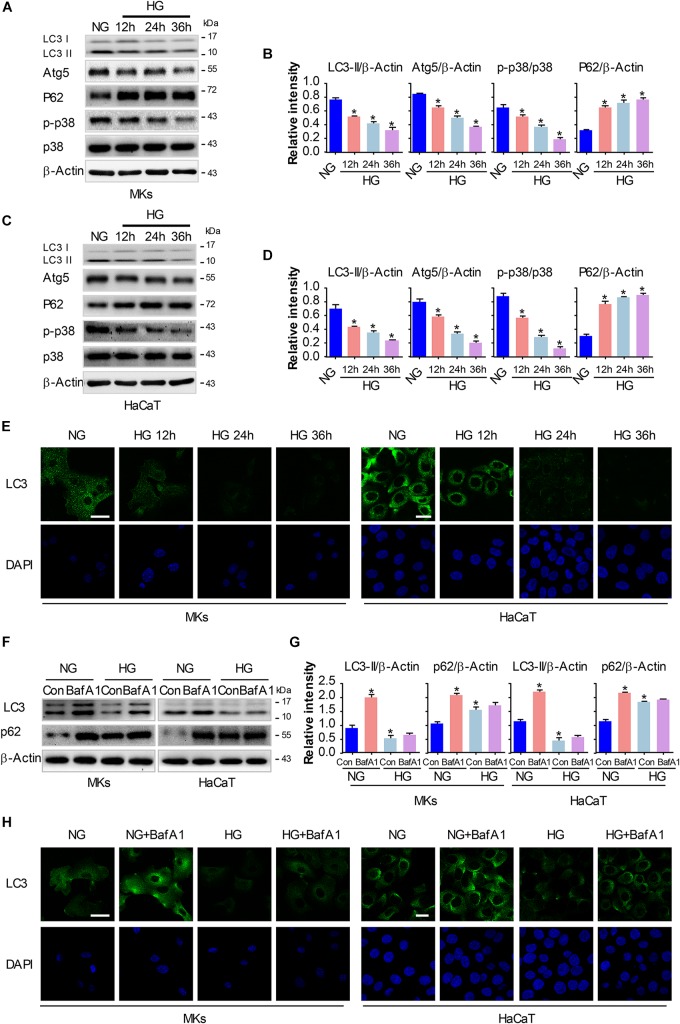
HG treatment inhibits autophagy and p38/MAPK activity in keratinocytes. MKs and HaCaT cells were subjected to 25 mM glucose and incubated for the indicated times (12, 24, and 36 h). The protein extracts were analyzed for the detection of autophagy and the activity of p38/MAPK using western blot. **(A,C)** Representative western blots were shown (*n* = 5). β-Actin was used as a loading control. **(B,D)** The graph represents the means ± SEM of the relative integrated signals. ^∗^*P* < 0.05 vs. NG group. **(E)** Fluorescence staining of LC3 expression (green) in the indicated keratinocytes was shown (*n* = 5). Nuclei were stained with DAPI. Scale bar = 25 μm. Then MKs and HaCaT cells were exposed to BafA1 (10 nM) for 1 h prior to HG treatment. The extracted proteins were immunoblotted with the indicated antibodies **(F)**. Representative western blots were shown (*n* = 5). β-Actin was used as a loading control. **(G)** The graph represents the means ± SEM of the relative integrated signals. ^∗^*P* < 0.05 vs. NG group. **(H)** Fluorescence staining of LC3 expression (green) in the indicated keratinocytes was shown (*n* = 5). Nuclei were stained with DAPI. Scale bar = 25 μm. All the experiments were repeated three times.

We and others have previously revealed that activation of p38/MAPK signaling was implicated in the enhancement of keratinocyte migration (Harper et al., 2005; Jiang et al., 2014). As a process of self-degradation that maintains cellular viability upon metabolic stress, autophagy has been reported to promote cell migration in some cases (Kenific and Wittmann, 2016). Moreover, recent studies suggest a role of p38/MAPK in regulating autophagy although there are controversies if it promotes or inhibits autophagy (Sridharan et al., 2011). To investigate the role of p38/MAPK signaling and autophagy in keratinocyte migration under HG treatment, we firstly evaluated the changes in p38/MAPK activation and autophagy under indicated treatments. MKs and HaCaT cells were subjected to 25 mM glucose for 12, 24 and 36 h. Results of western blot analysis showed that expression of phosphorylated p38 (p-p38) significantly decreased in keratinocytes under HG treatment, with the expression of p38 was unchanged, which was further confirmed by quantitative analysis of the ratio of p-p38 to p38 (Figure 2A–D). For the detection of autophagy, the expressions of Atg5, P62 and the conversion of LC3-I into LC3-II were analyzed in keratinocytes under indicated treatments (Figure 2A–D). Interestingly, keratinocytes under HG treatment showed significant reduction in the expressions of Atg5, P62 and autophagosome-associated pro-autophagic LC3-II as compared to the NG group. Moreover, a decrease of LC3 puncta, as monitored by confocal microscope, was found in keratinocytes under HG treatment (Figure 2E), which suggested the impairment of autophagosome formation.

To further investigate the autophagic flux in keratinocytes under HG treatment, we exposed keratinocytes to a lysosomal inhibitor bafilomycin A1 (BafA1, 10 nM). BafA1, a chemical inhibitor of vacuolar H+ ATPase (V-ATPases), inhibits lysosomal proton transport, blocks lysosomal acidification, and consequently inhibits autophagy (Maiuri et al., 2007). As shown in Figure 2F–H, LC3-II levels in keratinocytes exposed to BafA1 were significantly lower in HG compared with NG group, and similar results were found in immunofluorescence assay. Moreover, P62 expression was remarkably increased upon BafA1 treatment under NG group, but not so under HG condition, which suggested that the degradation process of autophagy was not impaired under HG conditions. Together, these observations revealed that HG treatment impaired autophagy and p38/MAPK activation in keratinocytes.

### P38/MAPK Signaling Regulates Autophagy and Migratory Capacity in Keratinocytes Under HG Treatment

**FIGURE 3:**
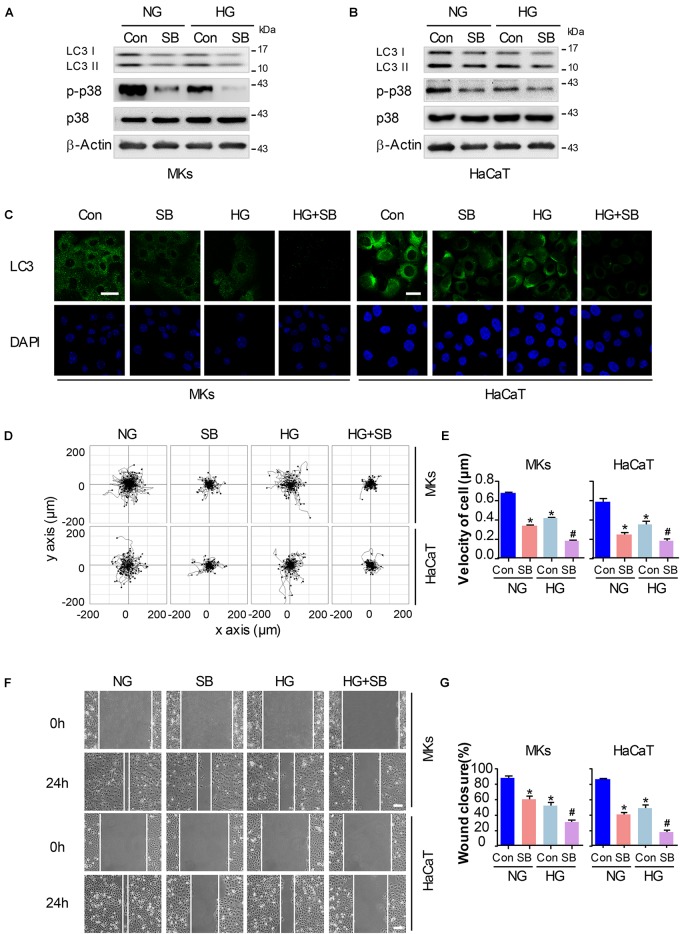
P38/MAPK signaling regulates autophagy and migratory capacity in keratinocytes under HG treatment. To explore whether the inactivation of p38/MAPK signaling is involved in the reduction of cell migration and autophagy in keratinocytes under HG treatment, a specific p38/MAPK inhibitor SB (5 μM) was used. **(A,B)** Western blot was performed to analyze the effects of SB on the activation of p38/MAPK signaling and autophagy. Representative bands were shown (*n* = 5). β-Actin was used as the loading control. **(C)** Fluorescence staining of LC3 expression (green) in the indicated keratinocytes was shown (*n* = 5). Nuclei were stained with DAPI. Scale bar = 25 μm. **(D)** Single cell motility assays were performed to detect the motility under HG (*n* = 5). Representative images of cell trajectories were shown. **(E)** Graph quantifying the average velocity of cell movement. Results were shown as means ± SEM. **(F)** Scratch wound healing assays were performed to detect the migration of indicated cells (*n* = 5). Pictures of the scratched wounding were taken after 24 h culturing with or without HG treatment. Representative pictures of the scratched wound were shown. Scale bar = 100 μm. **(G)** Graph quantifying the rate of wound closure. Results were shown as means ± SEM. ^∗^*P* < 0.05 vs. NG + Con group, ^#^*P* < 0.05 vs. HG + Con group. All the experiments were repeated three times. Con, control.

To explore whether the inactivation of p38/MAPK signaling involves in the decrease of cell migration and autophagy in keratinocytes under HG treatment, a specific p38/MAPK inhibitor SB (5 μM) was used. As expected, SB treatment suppressed the induction of autophagy and migration in keratinocytes of NG group. Interestingly, the addition of SB further decreased the expression of p-p38 and LC3 in keratinocytes when compared with HG group, as analyzed using western blot (Figure 3A,B and Supplementary Figure S5A). Moreover, results of fluorescence staining showed a significant decrease of LC3 puncta in keratinocytes (NG or HG group) when subjected to SB treatment (Figure 3C). Then the single cell motility assay and scratch wound healing assay were performed for the migratory detection of cells subjected to SB. Results showed a remarkable reduction in the range of cell trajectory and the velocity of cell movements in keratinocytes of NG group when exposed to SB, which was determined by single cell motility assay (Figure 3D,E). We also found a marked reduction of wound closure in the monolayer keratinocytes in NG group when exposed to SB as depicted with the scratch wound healing assay (Figure 3F,G). More importantly, the reduced migration of the keratinocytes under HG treatment was further decreased when subjected to SB (Figure 3D–G). These results suggested that p38/MAPK signaling was responsible for the regulation of the autophagy and migration in keratinocytes under HG treatment.

### Autophagy Regulates Keratinocyte Migration Under HG Treatment

**FIGURE 4:**
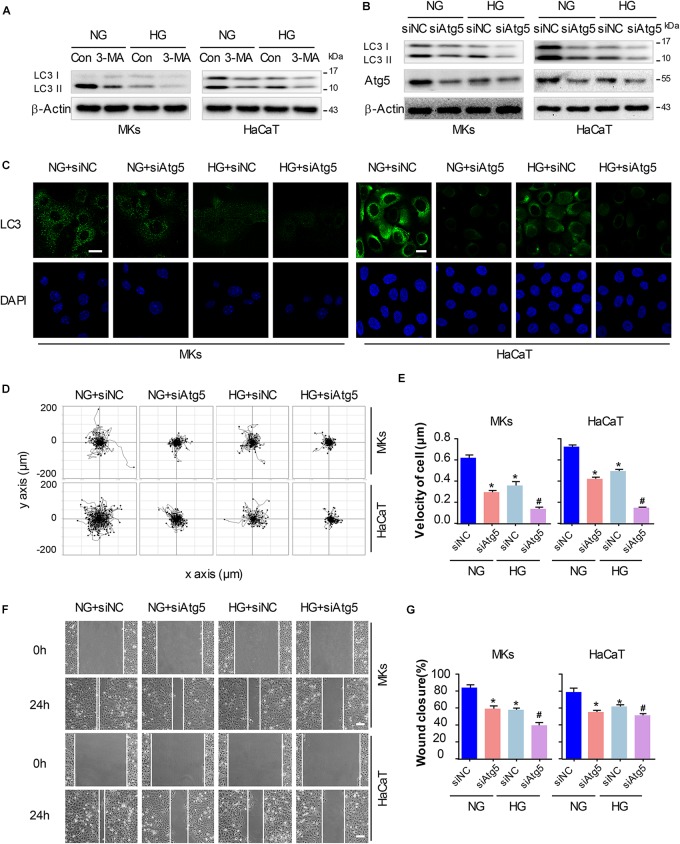
Autophagy regulates keratinocyte migration under HG treatment. To elucidate whether autophagy was involved in keratinocyte migration under HG treatment, 3-MA (5 mM) was applied to reduce the activity of autophagy in keratinocytes with or without HG treatment. **(A)** Western blot was performed to analyze the effects of 3-MA on the activity of autophagy (*n* = 5). Representative bands were shown. β-Actin was used as the loading control. To rule out the non-specific effects of chemicals and further confirm our results, cells were transfected with siRNA targeting Atg5 (siAtg5) or siRNA-negative control (siNC) before the treatment with or without HG. **(B)** Western blotting was used to analyze Atg5 and LC3 expression in the indicated keratinocytes (*n* = 5). Representative bands were shown. β-Actin was used as the loading control. **(C)** Fluorescence staining of LC3 expression (green) was performed in the indicated keratinocyte (*n* = 5). Nuclei were stained with DAPI. Scale bar = 25 μm. **(D)** Single cell motility assays were performed to detect the motility of indicated keratinocytes (*n* = 5). Representative images of cell trajectories were shown. **(E)** Graph quantifying the average velocity of cell movement. Results were shown as means ± SEM. **(F)** Scratch wound healing assays were performed to detect the migration of indicated cells (*n* = 5). Pictures of the scratched wounding were taken after 24-h culturing with or without HG treatment. Representative pictures of the scratched wound were shown. Scale bar = 100 μm. **(G)** Graph quantifying the rate of wound closure. Results were shown as means ± SEM. ^∗^*P* < 0.05 vs. NG + siNC group, ^#^*P* < 0.05 vs. HG + siNC group. All the experiments were repeated three times.

To elucidate whether autophagy was involved in keratinocyte migration under HG treatment, a chemical inhibitor, 3-MA (5 mM) was applied to reduce the activity of autophagy in keratinocytes with or without HG treatment. Results of western blot demonstrated that 3-MA remarkably inhibited the expression of LC3-II in keratinocytes under HG treatment, in which autophagy was impaired (Figure 4A and Supplementary Figure S5B). To rule out the non-specific effects of chemicals and further confirm our results, cells were transfected with siRNA targeting Atg5 (siAtg5) to suppress autophagy specifically, which was confirmed using western blot (Supplementary Figure S1). Atg5 was involved in the Atg12-Atg5 ubiquitin-like conjugation and lead to the elongation and sealing of the membrane of autophagosome. We found that specific knockdown of Atg5 in keratinocytes by siRNA transfection downregulated LC3-II expression, which indicated the inhibition of autophagy (Figure 4B and Supplementary Figure S5B). Furthermore, we performed immunofluorescence staining of LC3 by confocal microscope to confirm the effect of 3-MA and Atg5 knockdown on autophagy in keratinocytes under HG treatment. Results showed notable decreasing in LC3 puncta upon the exposure of Atg5 specific siRNA in keratinocytes with or without HG treatment (Figure 4C).

Basing on the results above, we explored whether autophagy regulated keratinocyte migration under HG treatment. The single cell motility assay and scratch wound healing assay were performed to monitor cell migration to appreciate its dependency on autophagy in indicated control cells and in cells with impaired autophagy, respectively. Results showed that Atg5 knockdown markedly decreased range of cell trajectory and the velocity of cell movement (Figure 4D,E). We also found that HG significantly reduced the migration of keratinocytes in the scratch wound model, and a significant wound gap was remained in the keratinocytes with Atg5 knockdown comparing with that keratinocytes treated with siRNA-negative control (siNC) (Figure 4F,G), which was in line with the results obtained from single cell motility assay. Similarly, chemical inhibition of autophagy by the addition of 3-MA also further impaired the migration of keratinocytes under HG treatment (Supplementary Figure S2). Together, these results indicate that autophagy is an important regulator of keratinocyte migration under HG treatment.

### P38/MAPK-Mediated Autophagy Involves in the Regulation of Keratinocyte Migration Under HG Treatment

**FIGURE 5:**
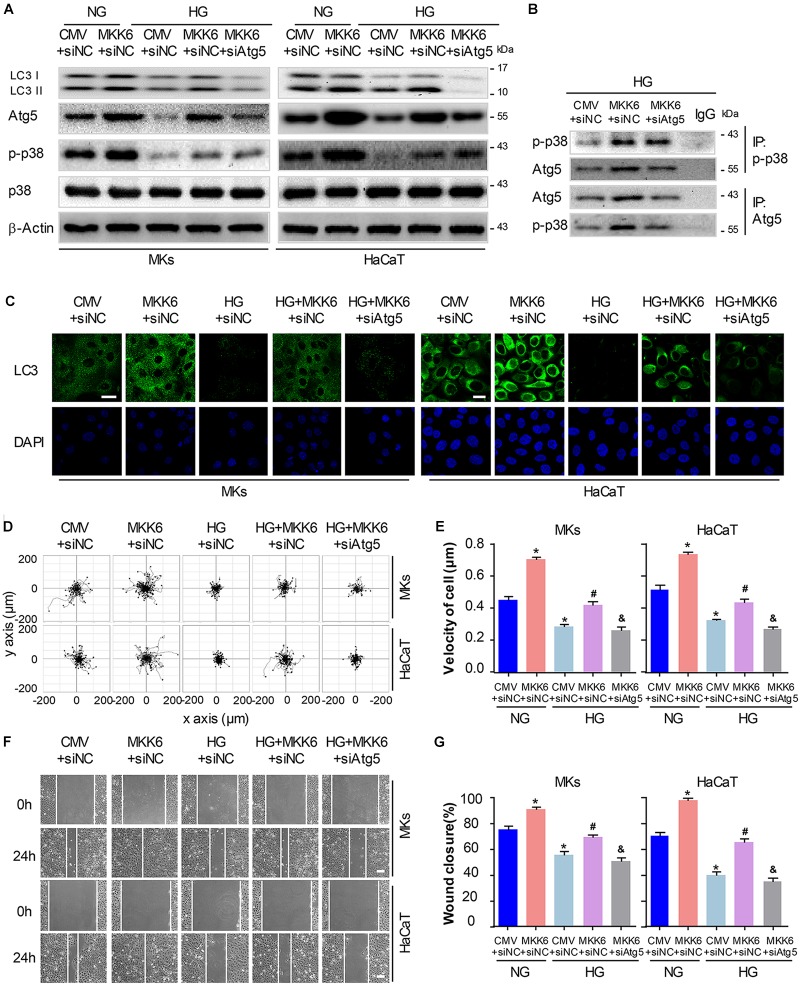
P38/MAPK-mediated autophagy involves in the regulation of keratinocyte migration under HG treatment. To gain further insights into the role for autophagy in the p38/MAPK-regulated keratinocyte migration under HG treatment, MKs and HaCaT cells were cotransfected with MKK6(Glu), siAtg5 or the corresponding negative control, CMV-null and siNC, before exposed to HG treatment. **(A)** Western blot was applied to detect the effects of transfection with MKK6(Glu), siAtg5 or both, on the autophagy of keratinocytes (*n* = 5). Representative bands were shown. β-Actin was used as the loading control. **(B)** The indicated cell lysates were prepared and immunoprecipitated with either agarose-conjugated anti-p-p38, anti-Atg5, or anti-IgG antibodies. Immunoprecipitates were analyzed by Western blot (*n* = 5). **(C)** Fluorescence staining of LC3 expression (green) was performed in the indicated keratinocyte (*n* = 3). Nuclei were stained with DAPI. Scale bar = 25 μm. **(D)** Single cell motility assays were performed to detect the motility of indicated keratinocytes (*n* = 5). Representative images of cell trajectories were shown. **(E)** Graph quantifying the average velocity of cell movement. Results were shown as means ± SEM. **(F)** Scratch wound healing assays were performed to detect the migration of indicated cells (*n* = 5). Pictures of the scratched wounding were taken after 24-h culturing with or without HG treatment. Representative pictures of the scratched wound were shown. Scale bar = 100 μm. **(G)** Graph quantifying the rate of wound closure. Results were shown as means ± SEM. ^∗^*P* < 0.05 vs. NG + CMV + siNC group, ^#^*P* < 0.05 vs. HG + CMV + siNC group, ^&^*P* < 0.05 vs. HG + MKK6 + siNC group. All the experiments were repeated three times. CMV, CMV-null. MKK6, MKK6(Glu).

To gain further insights into the role for autophagy in the p38/MAPK-regulated keratinocyte migration under HG treatment, we first constructed a MKK6(Glu) adenovirus to persistently activate MKK6, which induced the p38/MAPK activation, as confirmed by western blot analysis (Supplementary Figure S3). Then MKs and HaCaT cells were cotransfected with MKK6(Glu), siAtg5 or the corresponding negative control, CMV-null and siNC, before exposed to HG treatment. As shown in Figure 5A and Supplementary Figure S5C, in the keratinocytes under HG treatment, MKK6(Glu) notably stimulated the upregulation of the p38/MAPK signaling, and the expression of LC3-II, which were significantly attenuated by the cotransfection with siAtg5. Similar results were obtained in the immunofluorescence staining under confocal microscope (Figure 5C). It is noteworthy that activation of p38/MAPK signaling through MKK6(Glu) overexpression upregulated the expression of Atg5. We further investigated the interaction between p-p38, and Atg5. Results showed the interaction of p-p38, and Atg5 in HaCaT cells under HG treatment, which was significantly promoted with overexpression of MKK6(Glu), but was remarkably ameliorated after Atg5 was silenced (Figure 5B). These results suggested a molecular mechanism underlying the autophagy regulated by p38/MAPK signaling.

Next, the single cell motility assay and scratch wound healing assay were applied to investigate the migration of indicated keratinocytes. As shown in Figure 5D–G, overexpression of MKK6(Glu) showed a notable enhancement in the motility and migration of keratinocytes under HG treatment, while this effect was markedly abolished by the cotransfection with siAtg5. Taken with the above-mentioned results, activation of p38/MAPK signaling by MKK6(Glu) overexpression improved the attenuated keratinocyte migration under HG treatment through an autophagy-dependent mechanism, which suggested a role of autophagy in p38/MAPK-regulated cell migration.

### P38/MAPK Signaling and Autophagy Are Significantly Downregulated in Diabetic Mouse Epidermis

Treating animals with STZ leads to the occurrence of characteristic symptoms (polydipsia, polyphagia and polyuria) of diabetes. Diabetic mice showed a significant weight loss as compared to non-diabetic mice. Levels of blood glucose were significantly increased compared with controls. Pathologically, the epidermis of diabetic mice was thinner than those of non-diabetic mice (Supplementary Figure S4). Together, these results confirmed our model of DM to be successful.

**FIGURE 6:**
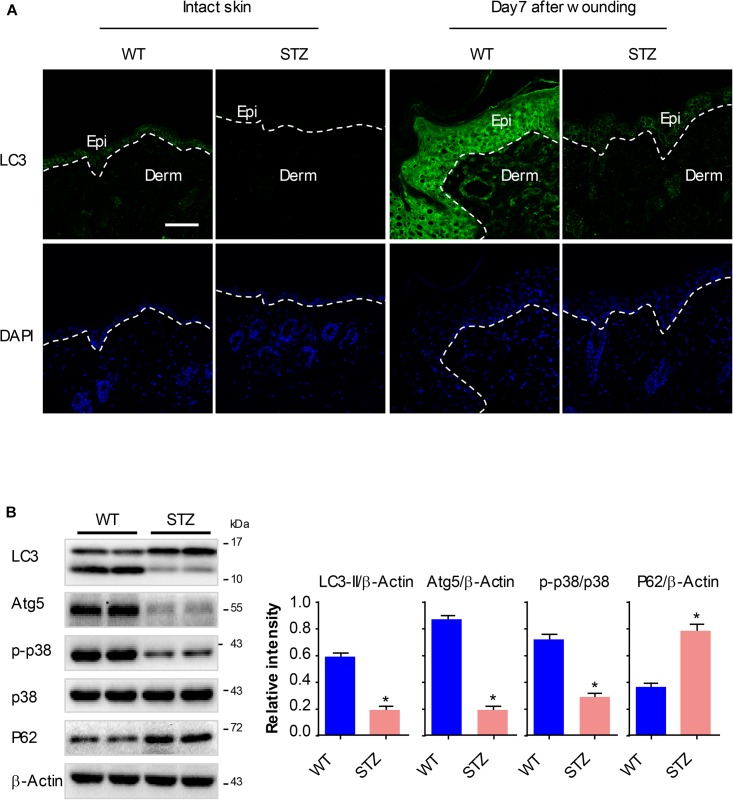
P38/MAPK signaling and autophagy are significantly downregulated in diabetic mouse epidermis. Full-thickness dorsal excisional wounds (5 mm in diameter) were made in STZ-induced diabetic and non-diabetic mice. The specimens of intact skin and wound were collected 7 days post wounding (*n* = 10). **(A)** Fluorescence staining of LC3 expression (green) was performed in the specimens of intact skin and wound. Nuclei were stained with DAPI. Scale bar = 75 μm. **(B)** Western blot was performed to analyze the expression profile of key proteins among p38/MAPK signaling and autophagy. Representative bands were shown. β-Actin was used as the loading control. Results of the quantitative analysis were shown as mean ± SEM. ^∗^*P* < 0.05 vs. WT group. WT, wild type.

To explore whether the p38/MAPK signaling and autophagy involve in diabetic wound healing, we analyzed the expression of key proteins using western blot and fluorescence staining analysis. Full-thickness dorsal excisional wounds (5 mm in diameter) in diabetic and non-diabetic mice. The wound specimens were collected 7 days post wounding. Expressions of LC3 were detectable in intact non-diabetic epidermis, but nearly not detectable in the diabetic epidermis. Upon wounding, the expression of LC3 was markedly increased in non-diabetic wounds compared with that in the intact non-diabetic wounds, while only a slight increase of LC3 was observed in the diabetic wounds (Figure 6A). Furthermore, results of western blot showed an impaired activation of p38/MAPK and an attenuated autophagy (as determined by the expression of Atg5, P62 and LC3-II) in diabetic epidermis compared with the non-diabetic epidermis (Figure 6B). These results demonstrated that p38/MAPK signaling and autophagy were consistently downregulated in diabetic mouse epidermis, which suggested a potential regulatory role for p38/MAPK in autophagy *in vivo*.

## Discussion

Impaired migration of keratinocytes results in the poor re-epithelialization during wound healing in patients with diabetes. In this study, we found that keratinocyte migration was impaired significantly under HG treatment, and its impairment was attributed to the inhibition of p38/MAPK and autophagy. More importantly, we demonstrated that overexpression of MKK6(Glu), an activator of p38/MAPK, rescued keratinocyte migration through an autophagy-dependent manner, thus uncovering a new pro-healing mechanism of the MAPKs, and simultaneously, providing a novel strategy for the clinical intervention of diabetic wounds.

Mitogen-activated protein kinases are important regulators to transduce the signaling of stress-induced stimuli or extracellular growth factor, and are crucial for the control of numerous cellular processes, such as migration, proliferation, and survival (Kim and Choi, 2010). We have previously found a marked activation of p38/MAPK pathway in the regenerated migrating epidermis *in vivo*, and demonstrated a promigratory role in hypoxic keratinocytes *in vitro* (Jiang et al., 2014). Growing evidences have revealed that activation of p38/MAPK signaling is associated with the complications arised from HG in patients with diabetes: p38/MAPK involves in HG-induced epithelial-to-mesenchymal transition (EMT) in human renal tubular cells (Song et al., 2018), in the HG-altered endothelial cell function (Alghamdi et al., 2018; Sáez et al., 2018), and in the apoptosis of podocytes (Taniguchi et al., 2013), all of which result in diabetic nephropathy. While few is known about the expression pattern and the role for p38/MAPK in diabetic wound healing. Here, we determined the activation of p38/MAPK in keratinocytes under HG using western blot analysis. We observed that p38/MAPK was notably downregulated in a time dependent manner upon HG stress. Consistently, an inhibition of p38/MAPK signaling was demonstrated in intact and regenerated wound epidermis of STZ-induced diabetic mice. SB, a specific p38/MAPK inhibitor showed a marked inhibition of keratinocyte migration under both NG and HG treatment. In the contrast, a constitutive p38/MAPK activator MKK6(Glu) was applied to stimulate the p38/MAPK signaling. We found that stimulation of p38/MAPK rescued the migratory capacities in keratinocytes under HG treatment. These results suggested that inhibition of p38/MAPK signaling might be responsible for the impaired keratinocyte migration under HG.

Then, an important issue raises from the molecular mechanisms underlying the p38/MAPK-regulated keratinocyte migration under HG. Here, we reported autophagy as a downstream target of p38/MAPK pathway. Increasing evidences suggest that autophagy plays an important role in cell migration, which is observed under different contexts (Kenific and Wittmann, 2016). As an important non-selective degradation machinery, autophagy is found to regulate the focal adhesion disassembly, β1 integrin membrane recycling, and the acquisition of mesenchymal markers, all of which are crucial for cell migration. While the precise positive or negative role for autophagy in cell migration varies depending on the cellular context (Tuloup-Minguez et al., 2013; Liu et al., 2017; Fontana and Vivo, 2018). To our knowledge, we firstly revealed the involvement of autophagy in the migration of keratinocyte under HG treatment. The observations here demonstrated that autophagy was downregulated in keratinocyte under HG; the inhibition of autophagy through a pharmacological (3-MA) or gene-targeted (Atg5 knockdown) way further worsened the already reduced keratinocyte migration; furthermore, the inhibition of autophagy by siAtg5 remarkably attenuated the increasing effects of MKK6(Glu) on keratinocyte migration. *In vivo*, we found that autophagy was downregulated consistently with the activity of p38/MAPK signaling in diabetic mouse epidermis. These observations indicated that inhibition of p38/MAPK signaling lead to the impaired keratinocyte migration under HG through an autophagy-dependent way. What is worth mentioning is the autophagy-independent mechanisms that involves in keratinocyte migration under HG condition according to previous literatures (Pan et al., 2015; Zhang et al., 2015; Long et al., 2016), such as ClC-2 chloride channels, NRF2, and FOXO1.

Another important issue is how p38/MAPK regulates autophagy in keratinocytes. Unexpectedly, we found that activation of p38/MAPK signaling through MKK6(Glu) overexpression upregulated the expression of Atg5, which is a key protein involving in the process of autophagy. As a protein kinase to regulate gene expression, p38/MAPK is very possible to regulate the transcription of Atgs. Further investigations revealed an interaction between p38 and Atg5, which helps to explain the role for p38 in regulating the expression of Atg5. Future studies are needed for the role of p38 MAPK in regulating other Atgs, which are also important in the induction of autophagy.

Together, our current work reveals a novel mechanism for the impaired migration of keratinocytes under HG. We showed that inhibition of autophagy resulted from the impaired activation of p38/MAPK signaling under HG was responsible for the poor migratory capacity of keratinocytes, which has been proved to cause delayed diabetic wound healing. These results reveal a novel molecular explanation for the impaired wound healing in situations where the levels of glucose are elevated, such as diabetes.

## Author Contributions

YH and JH supervised the work. LL, JuZ, and JH designed the experiments with help from JiZ. LL and JuZ performed the experiments with help from QZ, DZ, FX, JJ, and PW. JuZ and LL analyzed the data. LL, JuZ, and JH co-wrote the manuscript. All authors discussed the results and commented on the manuscript.

## Conflict of Interest Statement

The authors declare that the research was conducted in the absence of any commercial or financial relationships that could be construed as a potential conflict of interest.

## Footnotes

1 http://rsb.info.nih.gov/ij/
