# Supplementary material for: High Glucose Suppresses Keratinocyte Migration Through the Inhibition of p38 MAPK/Autophagy Pathway
Source: Front Physiol. 2019 Jan 28;10:24. doi: 10.3389/fphys.2019.00024 (PMC6360165; doi:10.3389/fphys.2019.00024)
Supplement: Supplementary file 1 [file Data_Sheet_1.docx]

**Supplementary Material**

**High glucose suppresses keratinocyte migration through the inhibition of p38 MAPK/autophagy pathway**

Lingfei Li^1,2*^, Junhui Zhang^1,2*^, Qiong Zhang^1,2^, Dongxia Zhang^1,2^, Jiezhi Jia^1,2^, Ping Wei^3^, Jiaping Zhang^4^, Liu Yao^5^, Jiongyu Hu^2,3*^, Yuesheng Huang^1,2*^

^1^Institute of Burn Research, Southwest Hospital, Third Military Medical University (Army Medical University), Chongqing, China.

^2^State Key Laboratory of Trauma, Burns and Combined Injury, Southwest Hospital, Third Military Medical University (Army Medical University), Chongqing, China.

^3^Endocrinology Department, Southwest Hospital, Third Military Medical University (Army Medical University), Chongqing, China.

^4^Department of Plastic Surgery, Southwest Hospital, Third Military Medical University (Army Medical University), Chongqing, China.

^5^Department of Pharmacy, Southwest Hospital, Third Military Medical University (Army Medical University), Chongqing, China.

Corresponding authors:

Jiongyu Hu, Endocrinology Department, State Key Laboratory of Trauma, Burns and Combined Injury, Southwest Hospital, Third Military Medical University (Army Medical University), Gaotanyan Street, Shapingba District, Chongqing 400038, China. Phone: +86 023 68773162. E-mail: jiongyuhu@163.com;

Yuesheng Huang, Institute of Burn Research, State Key Laboratory of Trauma, Burns and Combined Injury, Southwest Hospital, Third Military Medical University (Army Medical University), Gaotanyan Street, Shapingba District, Chongqing 400038, China. Phone: +86 023 68766023. E-mail: [yshuang1958@163.com](mailto:yshuang1958@163.com).

^*^These authors contributed equally to this work.

**
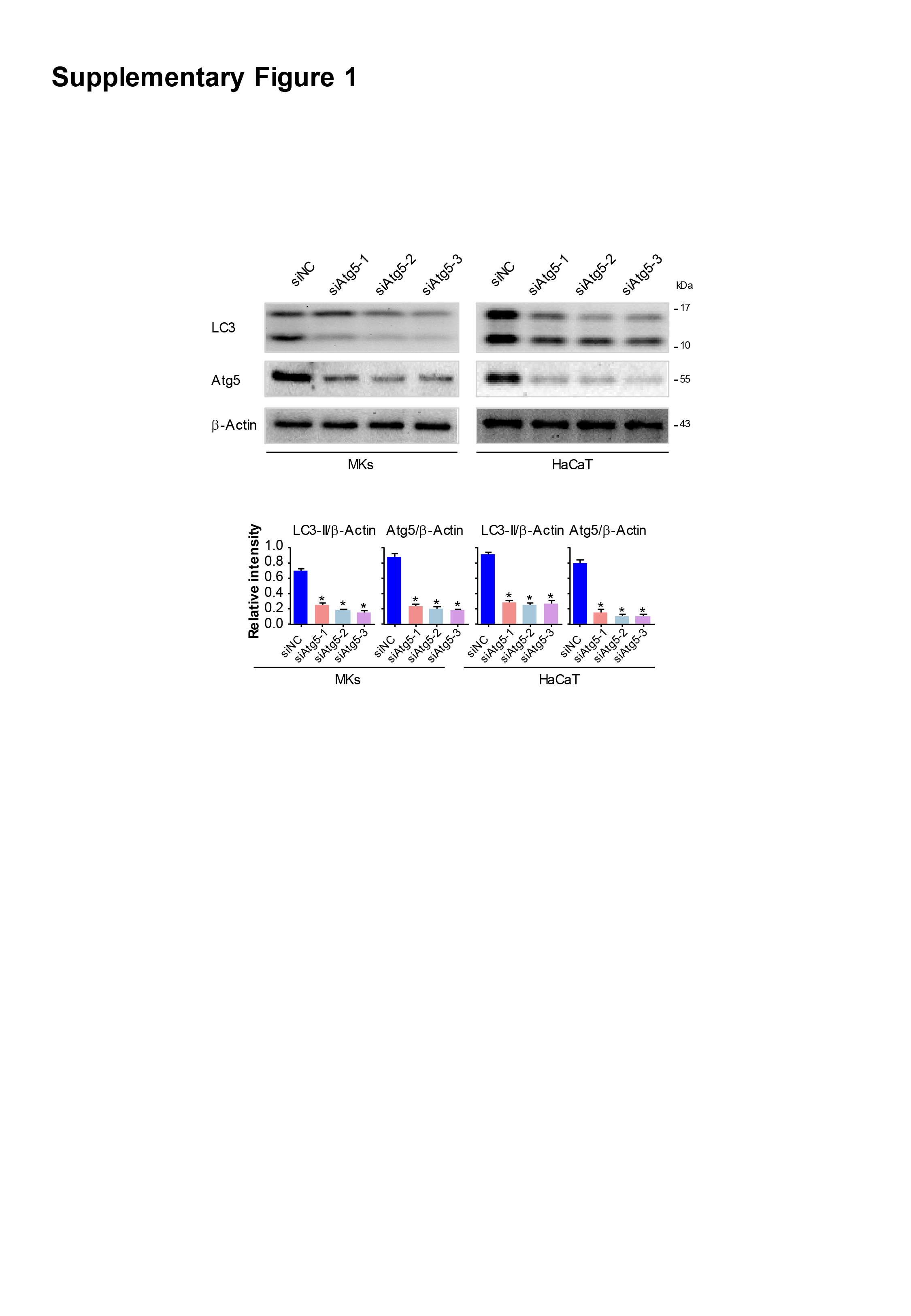
**

**Figure S1. Knockdown of Atg5 by siRNA transfection in keratinocytes.** Keratinocytes (HaCaT and MKs) were transfected with three different fragments of siRNA targeting Atg5 (siAtg5), or the siRNA-negative control (siNC) before the treatment with or without high glucose. Western blot was performed to detect the effectiveness of siRNA transfection (n = 5). Representative bands were shown. β-Actin was used as the loading control. The graph represents the means ± SEM of the relative integrated signals. ^*^*P* < 0.05 vs. siNC group. All the experiments were repeated 3 times.


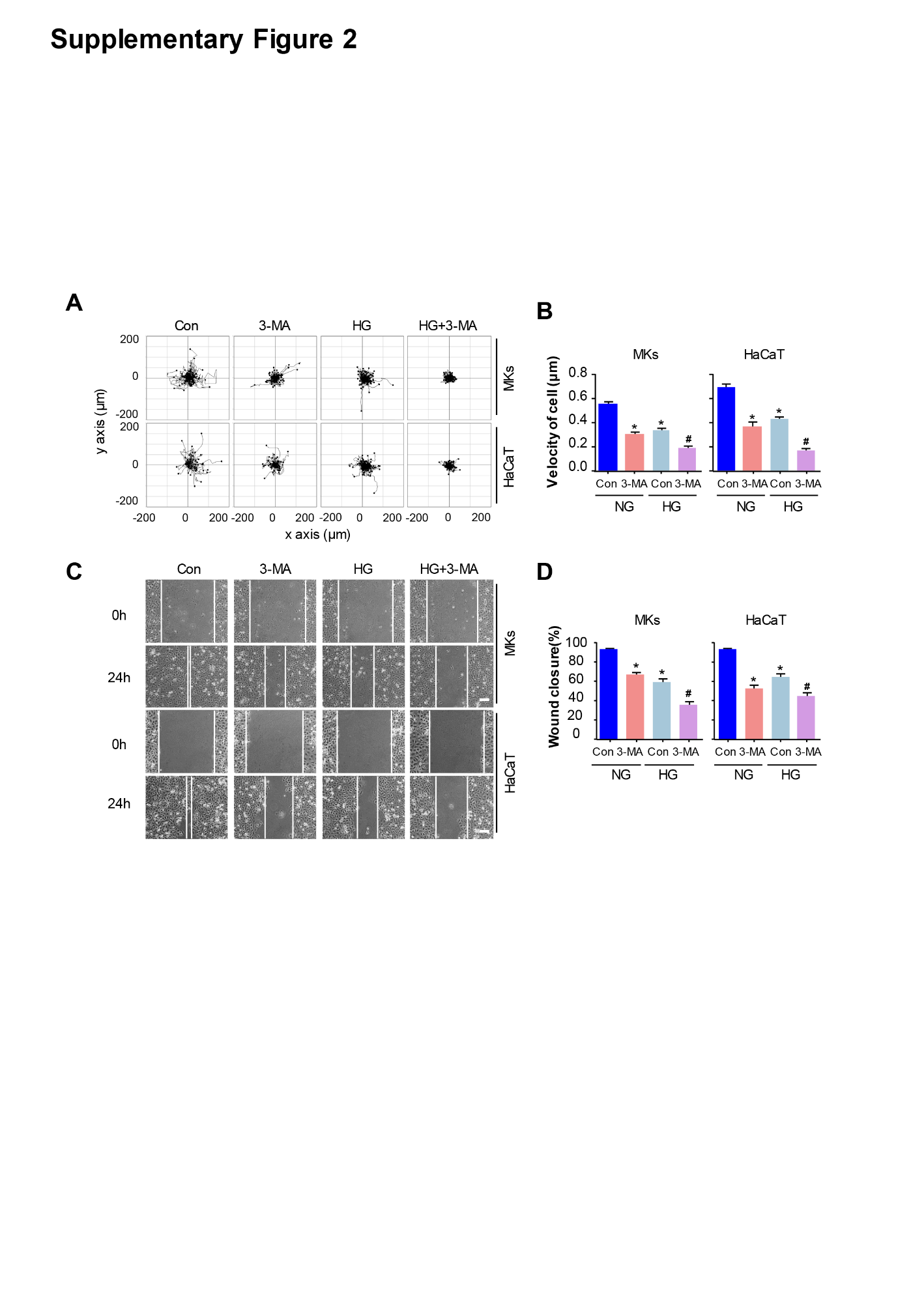


**Figure S2. 3-MA inhibited keratinocyte migration under high glucose treatment.** To elucidate whether autophagy was involved in keratinocyte migration high glucose treatment, 3-MA (5 mM) was applied to reduce the activity of autophagy in keratinocytes with or without high glucose treatment. **(A)** Single cell motility assays were performed to detect the motility of indicated keretinocytes (n = 5). Representative images of cell trajectories were shown. **(B)** Graph quantifying the average velocity of cell movement. Results were shown as means ± SEM. **(C)** Scratch wound healing assays were performed to detect the migration of indicated cells. Pictures of the scratched wounding were taken after 24-hour culturing with or without high glucose treatment (n = 5). Representative pictures of the scratched wound were shown. Scale bar = 100 μm. **(D)** Graph quantifying the rate of wound closure. Results were shown as means ± SEM. ^*^*P* < 0.05 vs. NG + Con group. ^#^*P* < 0.05 vs. HG + Con group.


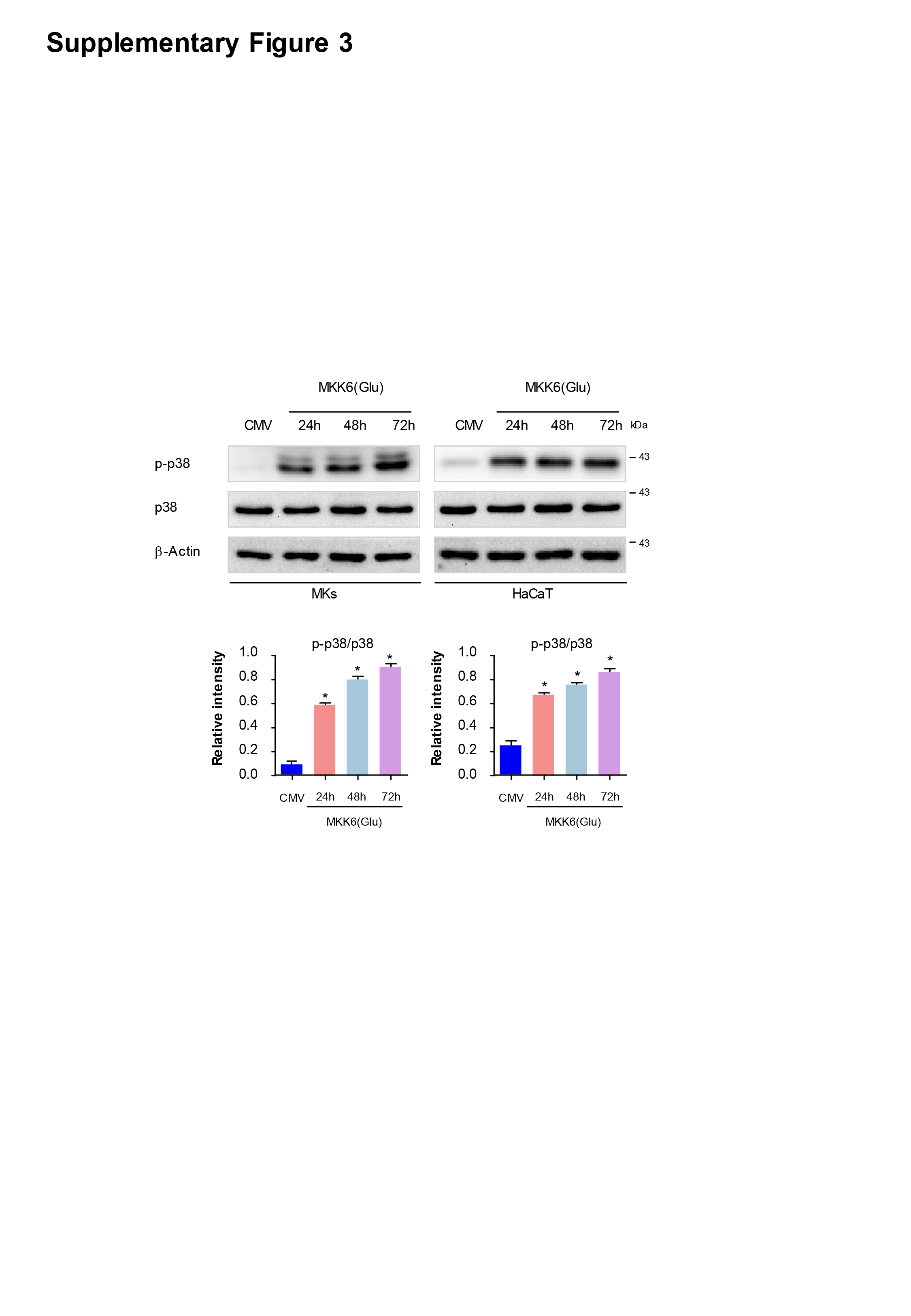


**Figure S3. Confirmation the effects of MKK6 (Glu) overexpression on p38/MAPK activation in keratinocytes.** We constructed a MKK6(Glu) adenovirus to persistently activate MKK6, which induced the p38/MAPK activation, as confirmed by western blot analysis (n = 5). The graph represents the means ± SEM of the relative integrated signals. ^*^*P* < 0.05 vs. CMV group.


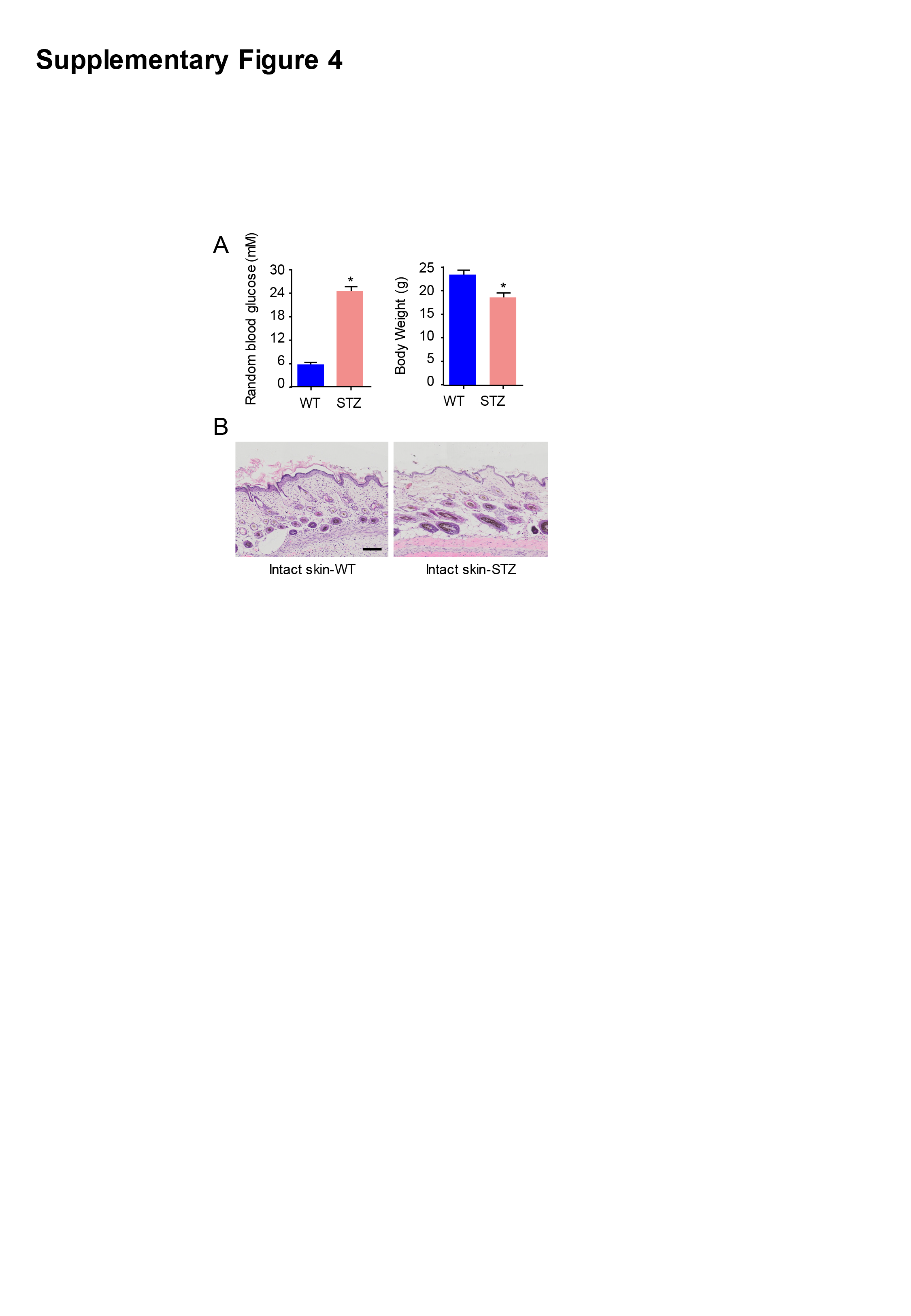


**Figure S4. Establishment of diabetic mouse model using STZ.** Mice were injected with a single intraperitoneal dose of STZ in saline at 150 mg/Kg body weight. **(A)** Body weight, random blood glucose were measured in a control group and STZ group. **(B)** Hematoxylin-Eosin staining of the intact skin from control group and STZ group. ^*^*P* < 0.05 vs. WT group. Scale bar = 100 μm.


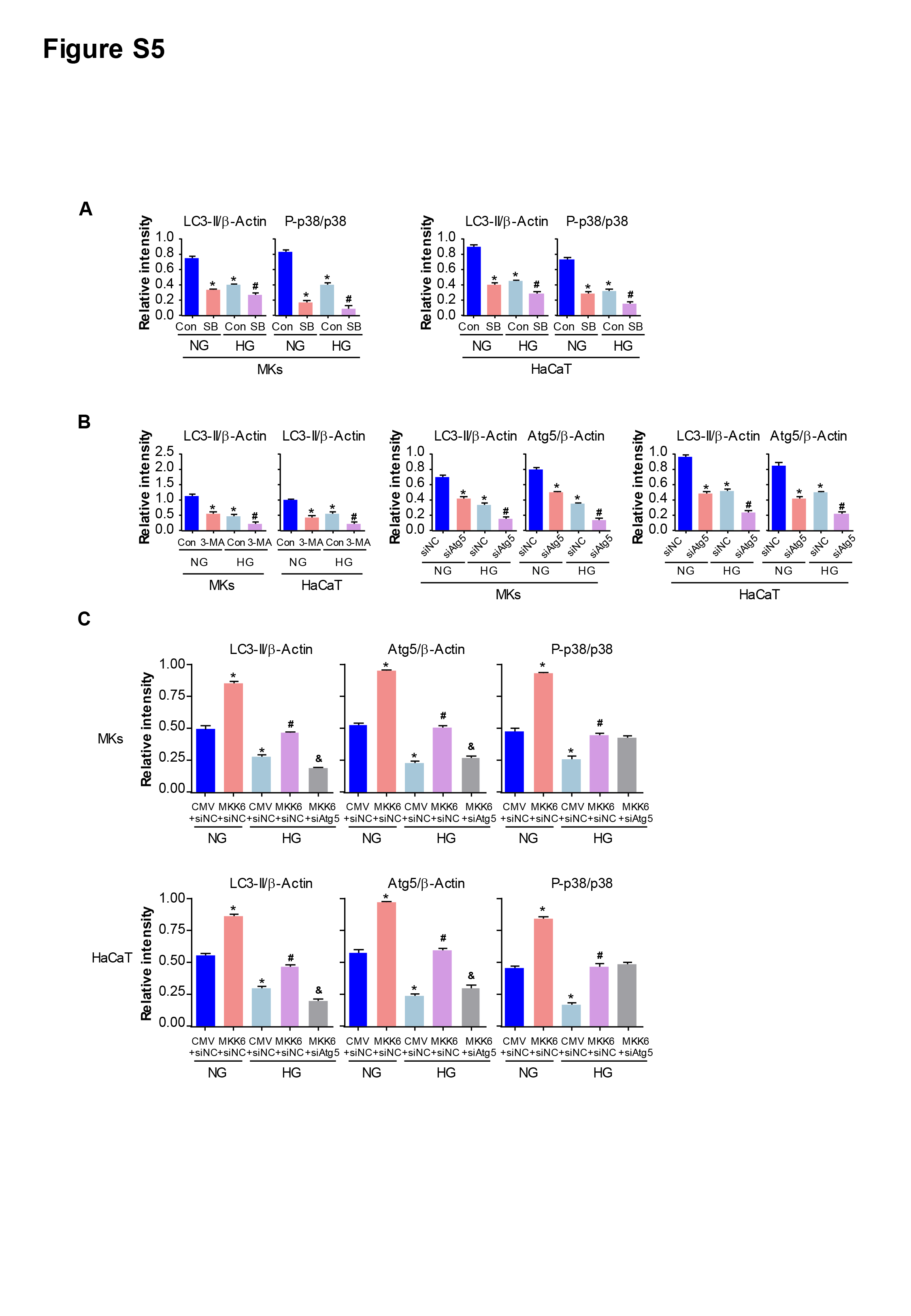


**Figure S5. Quantitative analysis of WB results.** WB results were quantitatively analyzed as corresponded to the results in Fig. 3-5. Fig. S5A-C was corresponded to Fig. 3A-B, 4A, and 5A, respectively. Data were shown as mean ± SEM (n = 5). ^*^P < 0.05 versus NG + Con group, ^#^P < 0.05 versus the HG+Con or HG + siNC or HG + CMV + siNC group, ^&^P < 0.05 versus HG + MKK6 + siNC group.
